# Protective Action of Spermine and Spermidine against Photoinhibition of Photosystem I in Isolated Thylakoid Membranes

**DOI:** 10.1371/journal.pone.0112893

**Published:** 2014-11-24

**Authors:** Hnia Yaakoubi, Saber Hamdani, Laurent Bekalé, Robert Carpentier

**Affiliations:** Groupe de Recherche en Biologie-Végétale, Université du Québec à Trois-Rivières, Trois-Rivières, Québec, Canada; University of Hyderabad, India

## Abstract

The photo-stability of photosystem I (PSI) is of high importance for the photosynthetic processes. For this reason, we studied the protective action of two biogenic polyamines (PAs) spermine (Spm) and spermidine (Spd) on PSI activity in isolated thylakoid membranes subjected to photoinhibition. Our results show that pre-loading thylakoid membranes with Spm and Spd reduced considerably the inhibition of O_2_ uptake rates, P700 photooxidation and the accumulation of superoxide anions (O_2_^−^) induced by light stress. Spm seems to be more effective than Spd in preserving PSI photo-stability. The correlation of the extent of PSI protection, photosystem II (PSII) inhibition and O_2_^−^ generation with increasing Spm doses revealed that PSI photo-protection is assumed by two mechanisms depending on the PAs concentration. Given their antioxidant character, PAs scavenge directly the O_2_^−^ generated in thylakoid membranes at physiological concentration (1 mM). However, for non-physiological concentration, the ability of PAs to protect PSI is due to their inhibitory effect on PSII electron transfer.

## Introduction

Plant productivity is highly dependent on the integrity of the photosynthetic apparatus. In the photosynthetic process, thylakoid membranes which are composed mainly of 4 supra-molecular protein complexes: photosystem II (PSII), photosystem I (PSI), cytochrome b_6_/f (Cyt b_6_/f) and ATP synthase. These complexes are responsible for the photochemical transformation of light energy into chemical energy resulting into the production of NADPH and ATP possessing high reducing power. Excitation of PSII with light produces electrons, protons and oxygen *via* water oxidation. The electrons are transferred to PSI through the transporters of the thylakoid membranes to reduce NADP^+^. In addition, the protons are used by ATP synthase for adenosine triphosphate (ATP) formation [1]. NADPH and ATP are then used to assimilate CO_2_ for sugar production *via* Calvin-Benson cycle.

However, excess light may affect photosynthetic processes such as the rate of electron-transport, oxygen evolution, and ATP production [2]–[6]. The damaging effect of light on photosynthetic activity is known as the photoinhibition process (PI). It is well established that the two photosystems may be targets for PI *in vivo* and *in vitro* under various environmental conditions. It was reported that PSI is more resistant at ambient temperature; the mechanism of this resistance is not yet well understood [7]–[13]. Although PSII is known to be more sensitive than PSI, it has been demonstrated that the inhibition of PSI is more deleterious to plants. This is not only due to the slow rate of its recovery but also due to its involvement in PSII repair as PSI is thought to provide the proton gradient required for ATP synthesis [14]–[16].

To avoid the strong damaging effects of light on photosynthetic membranes, plants have developed several protective mechanisms involving physical (alteration of leaf blade orientation), physiological (non-photochemical quenching) and biochemical changes (e.g, accumulation of enzymatic and non-enzymatic antioxidants) [5], [17]–[21]. Therefore, understanding these survival mechanisms presents a broad bearing on many fields including the development of photo-resistant plants. Nowadays, it is well known that oxidative stress constitutes the most important factor of the PI [22]–[24]. During this process, light, oxygen and electron transport induce the production of reactive oxygen species (ROSs) such as singlet oxygen (^1^O_2_), superoxide anion (O_2_^−^), hydrogen peroxide (H_2_O_2_) and hydroxyl radical (OH^.^). These species are mainly generated in PSI, but their damaging effects were observed even at the PSII level [15]. The oxidative stress may induce functional and/or structural modification of thylakoid membrane proteins to engender their damage [15], [22], [25], [26]. The most important line of defense against the oxidative stress implicates the enzymatic antioxidant system called water-water cycle [27]. This cycle is composed of several reactions which scavenge O_2_^−^ and H_2_O_2_. It involves the enzymes superoxide dismutase (SOD) and ascorbate peroxidase (APX), two enzymes located in the stroma and in the acceptor side of PSI [28], [29].

Recently, it has been observed that plants significantly increase the amount of various polyamines (PAs), such as spermine (Spm), spermidine (Spd) and putrescine (Put), under stress conditions [30]–[33]. For example, chilling temperatures can stimulate the S-adenosylmethionine decarboxylase, an enzyme implicated in PAs synthesis. The enhancement of PAs levels under stressful conditions may decrease plant susceptibility to photoinhibition [34]. Biogenic PAs are produced in the chloroplast and occur under free or conjugated forms [35], [36]. The conjugation of PAs is catalyzed by an enzyme named transglutaminase. This enzyme catalyses the incorporation of PAs into thylakoid and stromal proteins such as the light harvesting complex (LHC) and the large subunit of Rubisco [27], [38], [39]. Recently, Hamdani et al. [40] have investigated the beneficial role of some amines (Spm, Put and methylamine) as photo-protectors of PSII *in vitro*. It was found that only Spm showed photo-protective effect. This finding is considered important in understanding the mechanism of plant photo-adaptation in the presence of Spm. As PSI is also susceptible to photoinhibition under some conditions, it is reasonable to assume that PAs such as Spm could also exert a protective function against PSI photoinhibition. It is, therefore, of interest to determine the specific effect and to elucidate the photo-protective mechanism of these PAs.

The protective action of PAs against various stresses such as salt stress, UV-B radiation, ozone, heavy metal, or osmotic stress, is largely reported in the literature [41]–[45]. Most of these studies suggested that PAs protected plant cells *via* a direct interaction with their components or indirectly *via* its antioxidant role. However, the mechanism of their action is not yet fully understood. We provide here an insight on the mode of action of these PAs in protecting PSI activity in isolated thylakoid membranes. The measurements of electron transport, P700 photooxidation, and O_2_^−^ accumulation have been carried out to assess the effect of two PAs, with different positive charges and carbon chain, namely Spm and Spd on thylakoid membranes subjected to high light intensities. The structure of the two PAs is presented below:

^+^H_3_N – (CH_2_)_3_ – NH_2_^+^ – (CH_2_)_4_ – NH_3_^+^

**Spermidine**

^+^H_3_N – (CH_2_)_3_ – NH_2_^+^ – (CH_2_)_4_ – NH_2_^+^ – (CH_2_)_3_ – NH_3_^+^

**Spermine**

The results of this study showed that the activity of electron transport in PSI was significantly protected by the addition of these PAs even below 1 mM. The photo-protective effect increased with PAs concentration to reach a maximum at 7 mM. We also observed that the generation of O_2_^−^ in thylakoid membrane preparations was considerably reduced in the presence of the two PAs.

## Materials and Methods

### Thylakoid membranes isolation

Thylakoid membranes were isolated from fresh market spinach (Spinacia oleracea L.). Dark-adapted leaves were homogenized in a Tricine-NaOH buffer (50 mM pH 7.6) containing 400 mM sorbitol, 10 mM NaCl, 5 mM MgCl_2_ and 0.1% ascorbic acid. The suspension was filtered with miracloth tissue and centrifuged at 2550 g for 7 min. The pellet was then suspended in a same homogenization medium but without sorbitol. Then the pellet was washed with MES-NaOH solution (20 mM, pH 6.2) containing 15 mM NaCl, 10 mM MgCl_2_, and centrifuged for 7 min at 2550 g and 4°C. Finally, the pellet was resuspended in Hepes-NaOH buffer (20 mM, pH 7.6) containing 400 mM sucrose, 10 mM NaCl, 2 mM MgCl_2_ and 20 mM KCl [46]. The final thylakoid membranes preparation was kept in the dark, and chlorophyll (Chl) concentration was calculated following the procedure outlined in Porra et al. [47].

### Photoinhibitory treatment and polyamines addition

The samples of thylakoid membranes, at 500 µg Chl mL^−1^, were suspended in an assay medium containing 20 mM Hepes-NaOH (pH 7.6), 20 mM KCl, 10 mM NaCl and 2 mM MgCl_2_. Then, Spm or Spd solution was added at different concentrations. After few minutes, the samples were illuminated by an intense white light (2000 µmol of photons m^−2^ s^−1^) from a 150 W quartz-halogen projector lamp for 30 min with continuous stirring at 24°C controlled by water-bath.

### Oxygen uptake rates

The PSI activity was estimated by the measure of oxygen uptake rates in thylakoid membranes using an Oxylab system (Hansatech Instruments, Norfolk, England) at 24°C as described by Carpentier et al. [48]. The electrode chamber contained two compartments separated by a cellophane membrane. The activity of PSI was measured with DCPIPH_2_ (2,6-dichlorophenol indophenol) as electron donor and methyl viologen (MV) as a final electron acceptor. The assay medium was composed of the samples of thylakoid membranes pre- or un-illuminated, at a final Chl concentration of 10 µg mL^−1^, 20 mM Tricine-KOH (pH 7.8), 10 mM KCl, 10 mM NaCl, 5 mM MgCl_2_, 50 µM 3-(3,4-dichlorophenyl)-1,1-dimethylurea (DCMU), 500 µM MV, 1 mM NaN_3,_ 1 mM Na-ascorbate and 100 µM DCPIP. The reaction mixture was constantly stirred with a magnetic stirrer. After 1-min incubation, the reaction mixture was illuminated using a 150 W quartz-halogen projector lamp (500 µmol of photons m^−2^ s^−1^). The PSI activity was expressed in terms of µmol of oxygen consumed per mg Chl per hour (mg Chl^−1^ h^−1^).

### Oxygen evolvement rates

Oxygen evolution by PSII in thylakoid membranes was measured using an Oxylab system at 22°C. The samples were prepared in a buffer containing 20 mM MES-NaOH (pH 6.3), 1 mM NaCl, 0.5 mM MgCl_2_, 0.35 mM 2,6-dichlorobenzoquinone as a PSII electron acceptor. Thylakoid membranes pre-incubated or not with Spm or Spd were added to the buffer at the concentrations of 25 µg Chl mL^−1^. The reaction mixture was illuminated with saturating light and the rates of oxygen evolved were measured. The results were expressed as the percentage of PSII inhibition.

### P700 photooxidation

P700 photooxidation was monitored in thylakoid membranes as light-induced absorbance changes at 820 nm at room temperature using the dual wavelength emitter detector ED-P700DW connected via a PAM-101 fluorometer (Walz). The ED-P700DW unit detects strictly the differential absorbance changes between 810 and 860 nm peaking at 820 nm ascribed to the P700^+^ radical absorption and removes the plastocyanin absorbance changes. Far-red light (78 µmol m^−2^ s^−1^), that preferentially excites PSI was obtained by passing the beam from a Fiber-Lite light source (Microview, Thornhill, ON, Canada) through a RG-9 filter (Schott, Mainz, Germany). The assay medium contained 20 mM Hepes-NaOH (pH 7.6), 20 mM KCl, 10 mM NaCl, 2 mM MgCl_2_ and 500 µg Chl mL^−1^. The measurements were performed in the presence of 100 µM DCMU and 50 µM MV to avoid reduction of P700 by linear electron flow coming from PSII during measurements and to prevent charge recombination between P700^+^ and reduced acceptor side, respectively.

### Superoxide anions generation

The generation of superoxide anions was estimated by the reduction of nitro blue tetrazolium (NBT) according to Beauchamp and Fridovich [49]. The NBT photo-reduction assay was modified for use with photosynthetic membranes. Samples of thylakoid membranes pre-incubated or not with different Spm and Spd concentrations in Hepes-NaOH buffer (pH 7.6, containing 20 mM KCl, 10 mM NaCl and 2 mM MgCl_2_), were mixed with 0.5 mM NBT solution. Upon illumination of the mixture at 24°C the thylakoids generated the O_2_^−^ that reduces the NBT. The reduction of the yellow NBT resulted in its transformation into the purple formazan. This reaction is called O_2_^−^-dependent NBT reduction. An aliquot of the un-illuminated or illuminated preparation was diluted with the Hepes-NaOH buffer and the changes in optical density (OD) were followed at 560 nm. The increase in the rates of NBT photo-reduction reflected the accumulation of O_2_^−^ generated in the mixture.

## Results

### Protective effect of polyamines on photo-damage of photosystem I

#### 1. Effect of Spm and Spd on oxygen uptake rates

**Figure 1:**
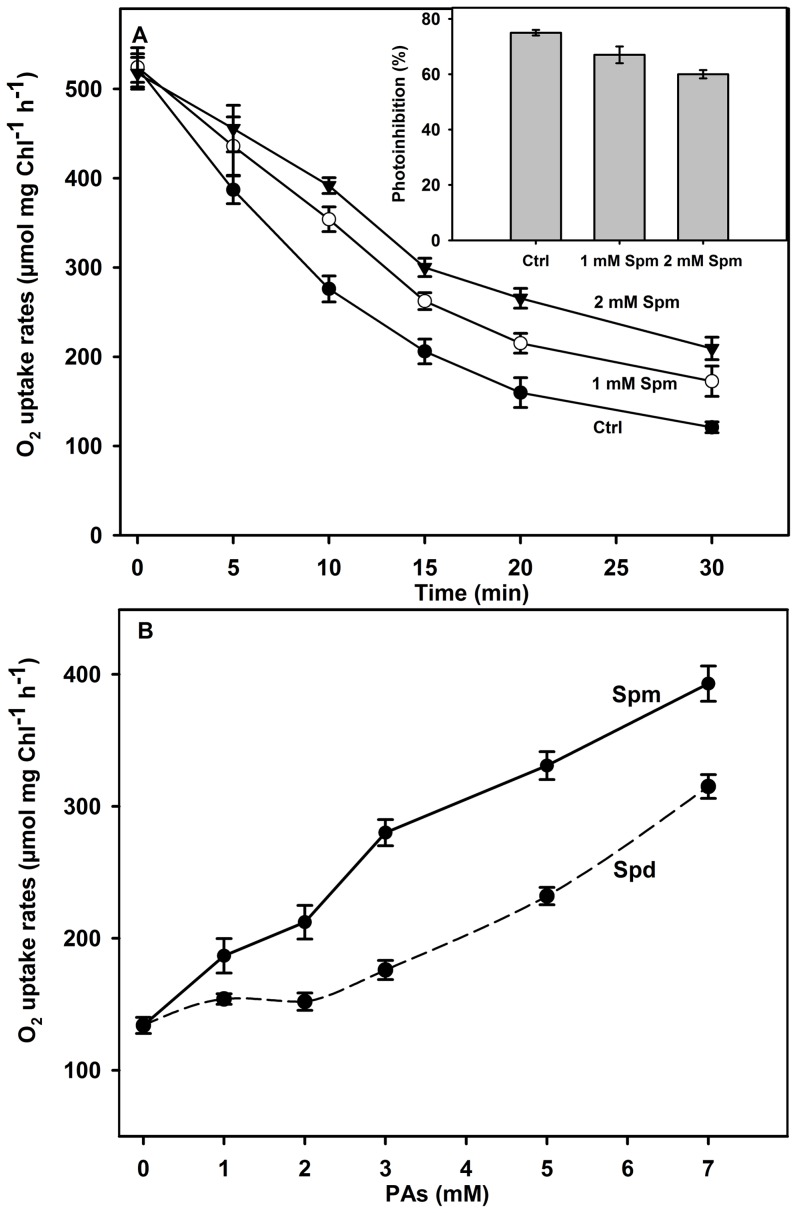
Changes in the O_2_ uptake rates in thylakoid membranes during 30 min of photoinhibition (A) in the absence (Ctrl) or presence of 1 or 2 mM Spm. Inset: percentage of photoinhibition in presence of 1 and 2 mM of Spm in thylakoid membranes, from the experiment of Fig. 1A. (B) Comparison of the effect of increasing the Spm and Spd concentration on O_2_ uptake rates in thylakoid membranes after 30 min of illumination. Control rate in the dark (Fig. 1A) was 525 µmol O_2_ mg Chl^−1^ h^−1^, and the results are a mean of 9 assays.

To investigate the effect of intense white light and two PAs on PSI activity, the oxygen uptake rates were measured in isolated thylakoid membranes exposed to strong illumination in the absence or presence of Spm or Spd. Reduced DCPIP was used as an artificial electron donor and MV as an electron acceptor. As shown in Fig. 1A (curve Ctrl), the initial oxygen uptake rate measured in control thylakoid membranes was estimated to 525 µmol oxygen consumed mg Chl^−1^ h^−1^. The rates of O_2_ uptake gradually decreased during the period of illumination. The loss of PSI activity was observed from the first minutes of irradiance and was reduced by 61% after 15 min of illumination and by 76% after 30 min. This inhibition of O_2_ uptake indicated the perturbation of electron transport through the thylakoid membranes and the dysfunction of PSI complexes [11], [50], [51]. Fig. 1A also shows the measured O_2_ uptake rates in thylakoid membranes preloaded with normal and supra-physiological concentrations of Spm (1 and 2 mM) and subjected to photoinhibition. The presence of Spm, a tetra-amine with four positive charges, reduced the inhibition of O_2_ uptake rates compared to photoinhibited control. After 30 minutes of illumination, the photoinhibition of PSI declined from 76% to 67% with 1 mM and to 60% with 2 mM (Fig. 1A, inset). The photo-protection was greater with 2 mM Spm than for 1 mM. It is clear that the addition of exogenous Spm in thylakoid membranes provided a protection to PSI against photoinhibition *in vitro*. This result prompted us to investigate the response of PSI activity to increasing Spm doses.

To determine the concentration dependence of Spm that protected PSI activity, O_2_ uptake rates were measured in samples photoinhibited in the presence of increasing doses of Spm. Surprisingly, the rates of O_2_ uptake remaining after photoinhibition greatly increased with Spm concentration (Fig. 1B). The maximal protective effect of Spm was reached at a non-physiological concentration, i.e, 7 mM. Above that dose, the rates of O_2_ uptake decreased (data not shown).

The comparison of the effect of Spm to that of Spd, a tri-amine with three positive charges (Fig. 1B), showed that Spd also protected the O_2_ uptake rates in a dose dependent manner (Fig. 1B). However, the protective action of Spd was not as strong as for Spm. The photo-protection of O_2_ uptake rates at 7 mM by Spm and Spd was near 70% and 50%, respectively.

It is interesting to note that the addition of Spm exerted a slight stimulatory effect on PSI activity in control samples even in dark (data not shown). This stimulation is generally significant for Spm concentrations above 3 mM and reached 16% at 7 mM Spm. Given that PSI activity is dependent on pH variation and the optimal activity is reached at about pH 9 [52], [53], the rise of pH in the thylakoid solution due to the presence of high doses of PAs increased PSI activity in control sample. Nevertheless, this Spm-induced stimulatory effect stayed much weaker compared to the photo-protective effect (Fig. S1).

#### 2. Action of Spm and Spd on P700 photooxidatio

**Figure 2:**
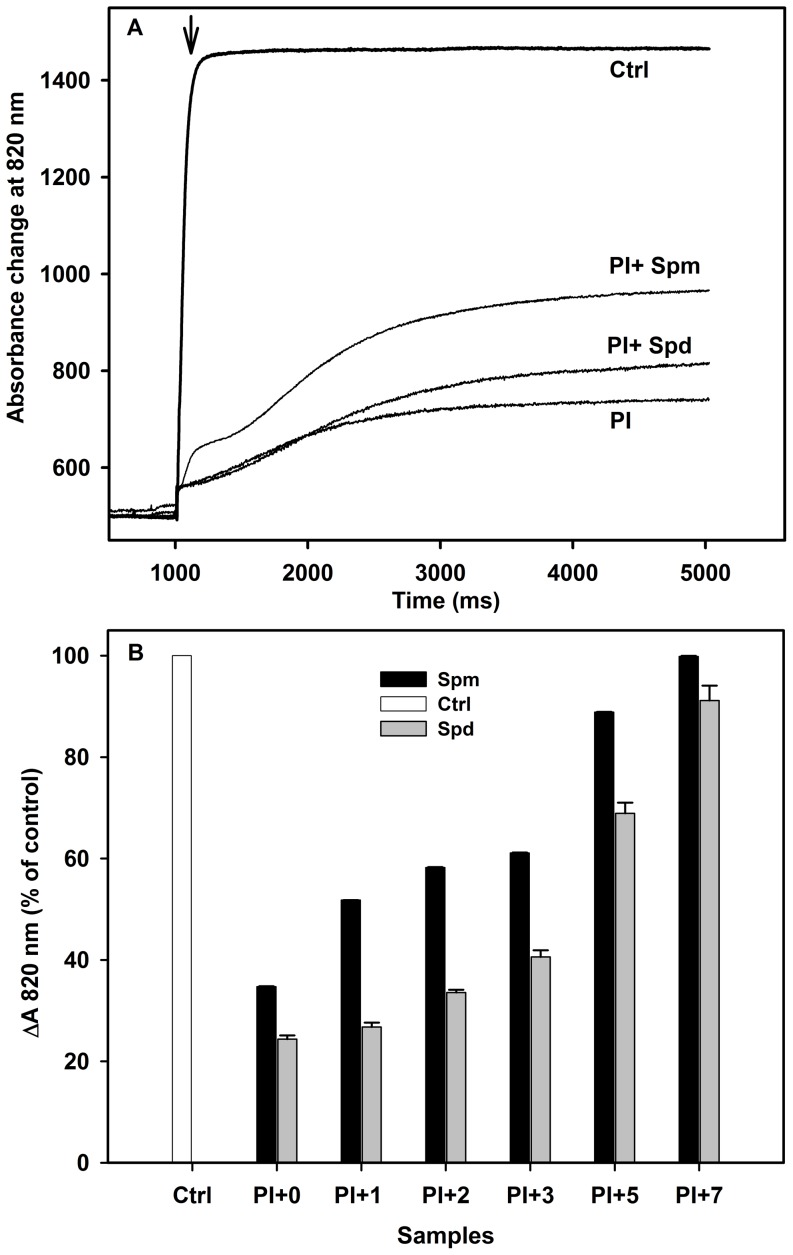
P700 photooxidation in thylakoid membranes after 30 min of photoinhibition. (A) Original traces of the Far-Red light-induced P700 photooxidation monitored as absorbance changes at 820 nm in the samples of thylakoid membranes either control (Ctrl) or photoinhibited for 30 min (PI) in the absence of PAs. PI+Spm and PI+Spd curves represent respectively, the absorbance changes at 820 nm in thylakoid membranes photoinhibited in presence of 1 mM Spm or Spd. The arrow indicates the switching on of Far-Red light (78 µmol m^−2^s^−1^). Each trace is the average of six measurements. (B) Variation of the amount of photooxidizable P700 (ΔA 820 nm) in the thylakoid membranes either control (Ctrl) or photoinhibited for 30 min (PI+0) in the absence of PAs. PI+1, PI+2, PI+3, PI+5 and PI+7 represent the percentage of photooxidizable P700 in photoinhibited samples in presence of varying concentrations (1–7 mM) of Spm and Spd. Each value is the average of six measurements.

To gain more information on the effect of strong white light and PAs (Spm and Spd) on PSI reaction centers, we analyzed the P700 photooxidation. The oxidation kinetics of P700 was measured in thylakoid membranes exposed to photoinhibition in the absence or presence of different concentrations of the two PAs (Fig. 2). The kinetics of the control sample (Fig. 2A, curve Ctrl) showed a fast initial photooxidation phase, which occurred within 0.65 s, leading to a prolonged steady-state phase. This curve represents the maximum photooxidizable P700 population obtained in the presence of DCMU and MV. When compared to the control, the photoinhibited samples (Fig. 2A, curve PI) showed a slower initial phase of photooxidation kinetics and the P700 oxidation reached its maximum after 3.7 s. The addition of 1 mM Spm increased significantly the rate of P700 photooxidation observed after photoinhibition (Fig. 2A, curve PI+Spm). The effect of Spd was less significant than that of Spm (Fig. 2A, curve PI+Spd).

Fig. 2B illustrates the amount of active P700 (ΔA 820 nm): the fraction that can be photooxidized. It shows a strong decrease of active P700 in photoinhibited samples (Fig. 2B, PI+0) compared to the control. It should be mentioned that photooxidizable P700 (%) in both PI+0 samples is supposed to be same, but the observed small variation is due to the use of different extracts of thylakoid membranes. However, increasing the doses of both Spm and Spd reduced the loss of active P700 compared to photoinhibited samples. Strikingly, at 7 mM Spm, almost whole population of photooxidizable P700 was preserved against the strong illumination (Fig. 2B, PI+7). In contrast, the protective effect of Spd on the level of P700^+^ cation radical increased slowly compared to Spm in the range between 1–3 mM. A strong protective effect was observed above 5 mM. As observed with the O_2_ uptake rates, Spm was more effective than Spd in protecting PSI reaction center under photoinhibitory treatment.

### Inhibition of O_2_^−^ generation by Spm and Spd

**Figure 3:**
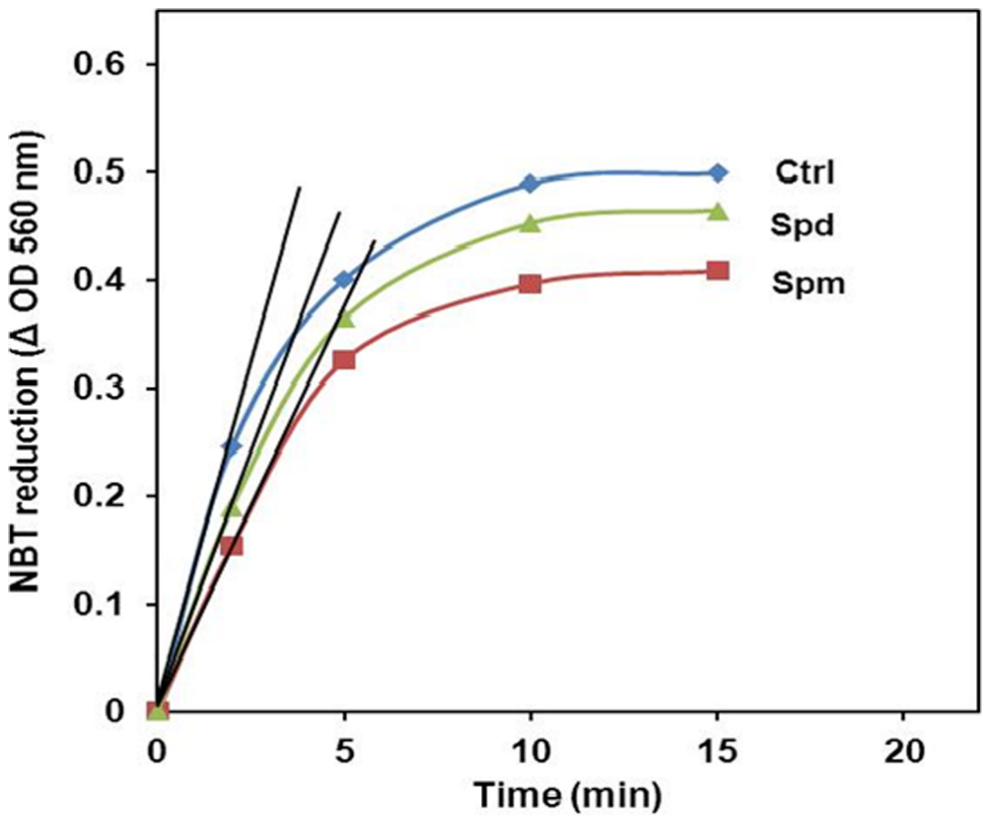
NBT photo-reduction in the absence or in presence of Spm and Spd. The changes in the optical density at 560 nm following NBT photo-reduction in thylakoid membranes preparation subjected to photoinhibition in the absence or presence of 1 mM Spm or Spd as a function of time.

The possible decrease in the generation of O_2_^−^ (the first precursor for the generation of H_2_O_2_ and OH^.^) at the PSI acceptor side in the presence of PAs was investigated using NBT as an O_2_^−^ chemical sensor. The measurements of NBT reduction, monitored as changes in optical density at 560 nm, were carried out for control or PAs-pretreated thylakoid membranes. It should be mentioned that a slight NBT reduction (OD 560 nm  = 0.010–0.015) was observed in the dark upon NBT addition to the thylakoid membranes incubated or not with PAs. The increase of OD 560 nm in the dark may be explained by a direct reduction of NBT by thylakoid membrane components *via* an O_2_^−^-independent pathway. This process is usually observed with biological membranes that contain some electron-transferring components that reduce NBT directly [49]. However, under illumination, the O_2_^−^-dependent NBT reduction strongly dominates. To determine the O_2_^−^-dependent NBT reduction, the values measured in the dark were subtracted from the values of the corresponding illuminated sample. The kinetics of NBT photo-reduction in thylakoid membranes photoinhibited either without (Ctrl) or with PAs (Spm and Spd) are presented in Fig. 3. Our results showed an enhancement of the NBT photo-reduction in the photoinhibited sample (Ctrl) with increasing irradiation time, indicating the generation of the superoxide anions. In the control sample, the superoxide anions are formed at an initial rate of 9.44×10^−2^ min^−1^. The rapid O_2_^−^ generation explained the loss of PSI activity in the thylakoid membranes under photoinhibition. However, when exogenous Spm (1 mM) was added to the thylakoid solution, the rate of O_2_^−^ generation was reduced from 9.44×10^−2^ to 6.99×10^−2^ min^−1^ (26%). Under the same conditions, 1 mM of Spd reduced the NBT photo-reduction rate by 13.5% (8.17×10^−2^ min^−1^). The inhibition of the NBT photo-reduction by PAs revealed their O_2_^−^ scavenging character. This result also indicates that Spm is more effective in reducing the O_2_^−^ accumulation than Spd.

### Correlation between PSI photo-protection, PSII inhibition and O_2_^−^ generation by Spm and Spd

**Figure 4:**
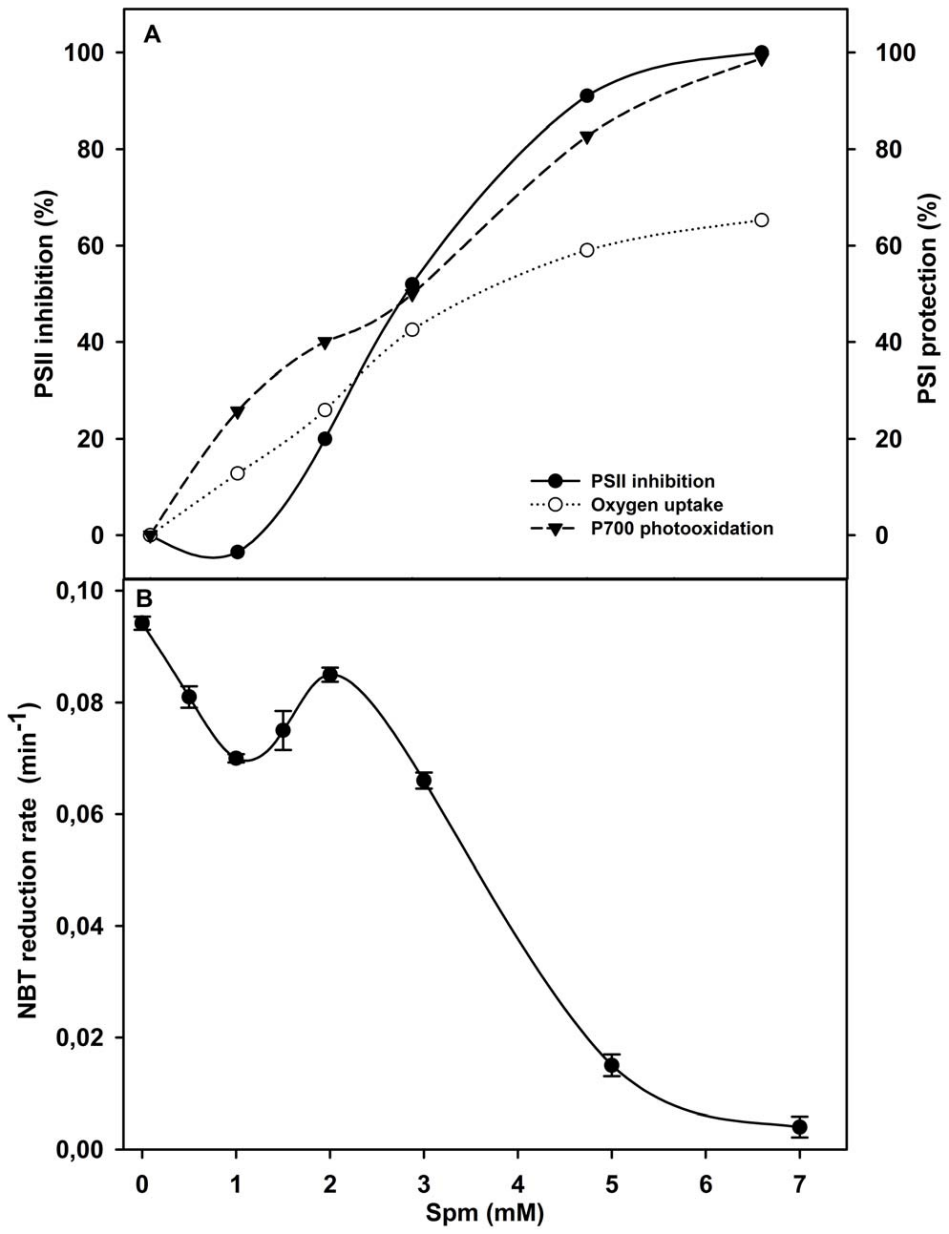
Correlation of the PSI protection, PSII inhibition and NBT-photo-reduction in the presence of Spm. (A) Effect of increasing Spm doses on (i) inhibition of PSII in control thylakoid membranes (in dark) and (ii) protection of PSI activity after 30 min photoinhibition, expressed as percentage of P700 photooxidation and O_2_ uptake obtained from the experiments of Fig. 1B and Fig. 2B and calculated using the following formula: 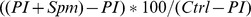 Were PI+Spm is the photoinhibited sample in presence of Spm, PI is the photoinhibited samples and Ctrl is the control. (B) The variation of the initial rate of NBT photo-reduction in thylakoid membrane preparations subjected to photoinhibition in the absence or presence of increasing Spm doses. The rates were obtained as described in Fig. 3.

Given that Spm and Spd affect PSII electron transfer in PSII sub-membrane fractions [54], their effect on PSII activity in thylakoid membranes and their consequences on PSI photo-protection were also studied. To estimate the effect of Spm on PSII activity, thylakoid membranes were incubated with different PAs concentrations in the dark and the inhibition of PSII activity is shown in Fig. 4A. The response of PSII shows that the normal Spm dose (1 mM) did not exert a negative effect on the PSII photochemical activity but, in the contrary, it slightly increased the PSII activity (≈5%). However, when the added Spm exceeded 1.5 mM, PSII inhibition progressively increased and the total inhibition was observed at about 6–7 mM.

The correlation of PSII inhibition and PSI protection as expressed in terms of P700 photooxidation and O_2_ uptake with Spm is illustrated in Fig. 4A. The results allowed us to differentiate two phases with regard to inhibition of PSII activity. The first one is observed for doses below 1.5 mM where the PSII activity is not greatly affected while the PSI photo-protection readily increases. This effect of Spm on PSI activity may be ascribed to the O_2_^−^ scavenging action as observed by the decrease of the rate of O_2_^−^ production (Fig. 3). The second phase is observed for the concentrations higher than 1.5 mM Spm and may be divided into two parts: (i) for doses between 1.5–3 mM, the loss of PSII activity and the protection of P700 photooxidation increased, but the latter stayed more prominent than the PSII inactivation, (ii) for Spm doses higher than 3 mM, the inhibition of PSII electron transport and the protection of P700 photooxidation were closely similar. On the other hand, the protection of PSI activity measured as O_2_ uptake rates also increased with the Spm added doses, but not to the same extent as P700 oxidation. These results suggest that Spm, depending in its concentration, can protect PSI against PI by two different mechanisms: scavenging O_2_^−^ and by regulating PSII electron transfer.

To better estimate the Spm concentration range for these mechanisms, we measured NBT photo-reduction to determine the O_2_^−^ generated in the presence of different Spm doses (Fig. 4B). The results show that the initial rate of O_2_^−^ production displayed a complex response depending on the concentration of Spm. When the doses of Spm were below 1 mM, a significant decrease in the rate of NBT reduction was observed. After that, the addition of 1.5–2 mM Spm to the thylakoid preparation increased the rate of NBT reduction, but it did not surpass the control rate. Surprisingly, at Spm doses higher than 3 mM, the rate of NBT reduction decreased sharply and correlated well with PSII inhibition in dark (Fig. 4A). This indicates that at these Spm concentrations the decrease of NBT reduction rate is due to the inhibition of electron transfer from PSII to PSI by Spm. It is likely, that the concentration of Spm in the range of 1.5–2 mM constitutes a transient phase between its roles as antioxidant and as a regulator of PSII electron transfer.

**Figure 5:**
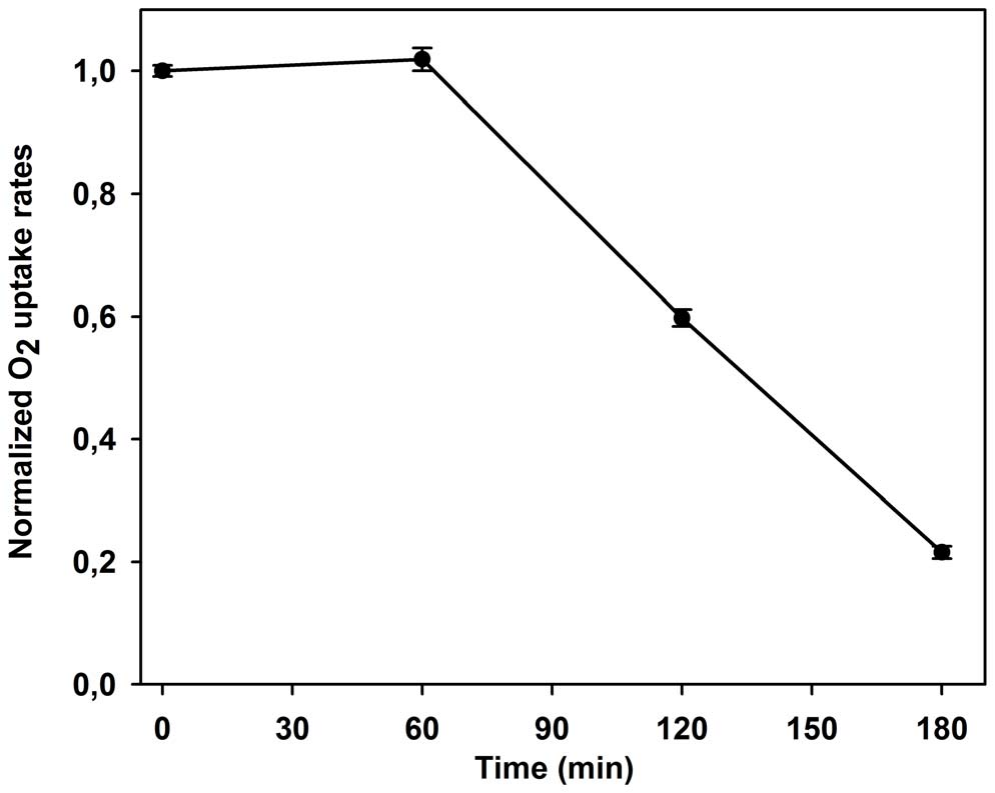
Photo-protection of PSI activity by DCMU. The measurement of O_2_ uptake rates in thylakoid membranes treated with 1 mM DCMU and exposed to photoinhibition for 180 minutes. The results are expressed as normalized values of O_2_ uptake rates.

To confirm the implication of decreasing PSII electron transfer in PSI photo-protection, we measured the O_2_ uptake rates in DCMU treated thylakoid membranes. Our results (Fig. 5) demonstrate that, the total inhibition of electron transfer from PSII to PSI by DCMU fully preserved PSI activity (O_2_ uptake rates) during the first hour of exposure to strong light. The decrease of the O_2_ uptake was observed only after prolonged period of illumination (120–180 minutes). The comparison of this result with the loss of PSI activity in the absence of DCMU (Fig. 1A, curve Ctrl) clearly demonstrates that the flux of electrons from PSII constitutes a prerequisite condition for PSI inhibition.

**Figure 6:**
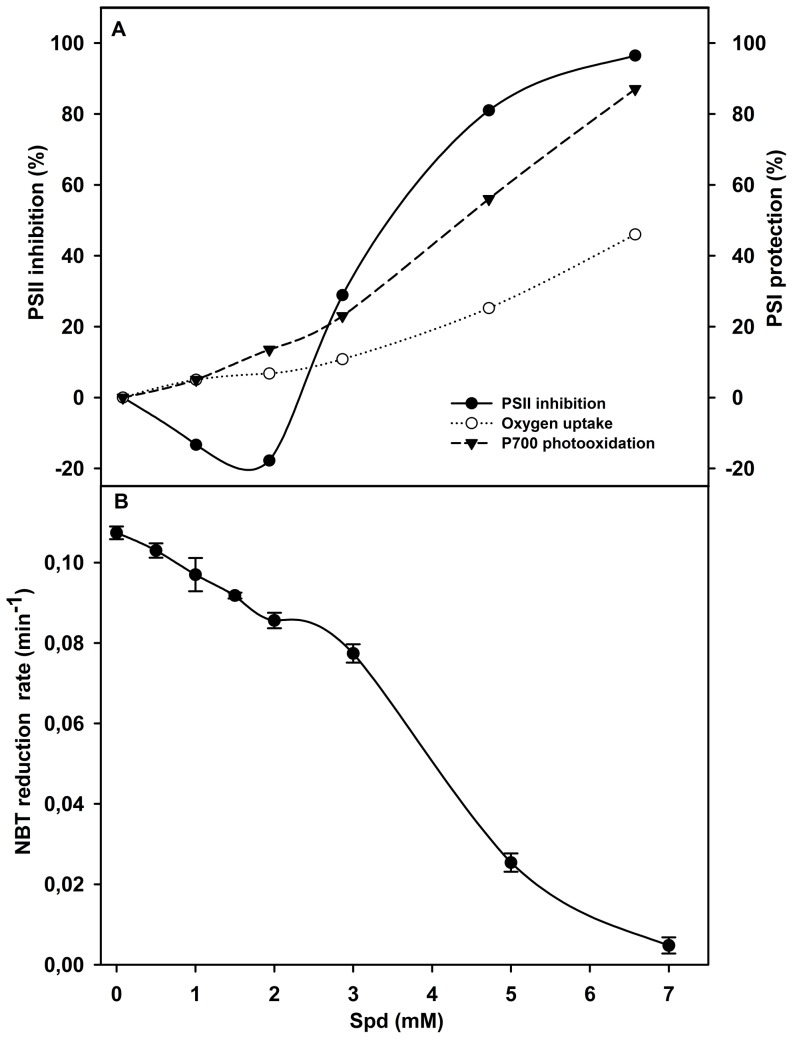
Correlation of the PSI protection, PSII inhibition and NBT photo-reduction in presence of Spd. (A) Effect of increasing Spd doses on (i) inhibition of PSII in control thylakoid membranes (in dark) and (ii) protection of PSI activity after 30 min photoinhibition, expressed as percentage of P700 photooxidation and O_2_ uptake obtained from the experiments of Fig. 1B and Fig. 2B, and calculated using the following formula: 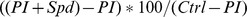 Were PI+Spd is the photoinhibited sample in presence of Spd, PI is the photoinhibited samples and Ctrl is the control. (B) The variation of the initial rate of NBT photo-reduction in thylakoid membrane preparations subjected to photoinhibition in the absence or presence of increasing Spd doses. The rates were obtained as described in Fig. 3.

In the case of thylakoid membranes treated with Spd, the inhibition of PSII activity (Fig. 6A, curve PSII inhibition) showed a generally comparable response to that of Spm (Fig. 4A, curve PSII inhibition). Up to 2.5 mM, Spd induced a greater stimulation of PSII activity (20%) than Spm. It is known that PAs are associated with the components of the thylakoid membranes and are considered as regulators of the photosynthetic processes [43]. The activation of PSII electron transfer with addition of low PAs concentrations to thylakoid membranes (Fig. 4A and 6A) showed that the microenvironment was changed. One plausible explanation for this stimulation is the interaction of added PAs with photosynthetic membranes lacking their associated PAs as by the extraction procedure. This interaction could allow the restoration of the appropriate protein structural organization for electron transfer. At 2.5 mM, the protection of P700 oxidation and O_2_ uptake did not exceed 12% and 7% respectively. The inhibition of the electron transfer in PSII was observed only above 2.5 mM, and increased rapidly to reach maximum (95%) at 7 mM. However, the protection of P700 oxidation increased progressively with Spd concentration (85% at 7 mM). The response of the O_2_ uptake rates to increasing Spd concentrations was less important than that of P700 oxidation (Fig. 6A) as for Spm. This is probably due to the sensitivity of some membrane-bound electron carriers to the higher dose of polyamines. On the other hand, the rate of NBT reduction decreased slowly at first between 0.5–3 mM Spd, but then rapidly decreased for doses higher than 3 mM (Fig. 6B). Our results demonstrate that the mechanisms of the photo-protection of PSI activity in thylakoid membranes are similar for both Spm and Spd, and that they depend on the PAs concentration.

## Discussion

In the present work, we provide some evidence of the protective action of Spm and Spd on the PSI activity in thylakoid membranes under photoinhibitory conditions. This protection was observed when Spm and Spd were added at known physiological concentration (1 mM) and also at higher doses of PAs. The potential mechanisms implicated in the photo-protection of PSI activity are discussed below.

In this study we have shown that high light intensity affected rapidly the activity of electron transfer in PSI under *in vitro* conditions as measured by the decrease of O_2_ uptake rates (Fig. 1A). The alteration of PSI activity by PI includes the decrease of the electron transfer from the donor side of PSI (Cyt b_6_/f and PC) to its acceptor side [11], [12], [55], [56]. In photoinhibited PSI sub-membrane fractions, Hui et al. [12] associated the initial fast PSI inhibition to the detachment of the LHCI antenna. They considered the loss of the peripheral LHCI_680_ antenna as a photo-protective mechanism that decreased excess energy transfer to PSI core. The important decline of PSI activity was observed at the end of treatment (Fig. 1A at 30 min). At this stage, the inhibition of O_2_ uptake is associated to a slow rate of P700 photooxidation and the loss of its active forms as observed in Fig. 2. This latter perturbation reflects the breakdown of the PSI reaction center (P700) that constitutes a common feature of PSI photoinhibition [11], [12], [57]. Moreover, the investigation of the mechanisms of PSI photo-inactivation relates its dysfunction to the degradation of the subunits of the acceptor side mainly the PsaC, PsaD, and PsaE and/or the reaction center proteins (PsaA and PsaB) [8], [15], [24], [50], [51].

The above functional and structural perturbations of the PSI complex are known to be part of a photo-oxidative process [8], [57]. Our results support the above idea as we indeed demonstrated that O_2_^−^ generation (Fig. 3) was concomitant with the loss of PSI activity (Fig. 1A, curve PI). However, the presence of Spm and Spd in the thylakoid preparation provided a scavenging effect against O_2_^−^ (Fig. 3). We suggest that exogenous Spm and Spd can improve the antioxidant defense system reducing thereby PSI inhibition (Fig. 1 and 2).

It is known that exogenous PAs can prevent the lipid peroxidation in photosynthetic membranes and stabilize their proteins like cytochrome f, plastocyanin, PSII manganese-stabilizing protein and D1/D2 proteins against different stress conditions [32], [45]. Generally, the generation of photo-oxidative stress in the photosynthetic membranes under strong illumination follows the dysfunction of the antioxidant defense system. Indeed, the antioxidant enzymes located near or at the PSI acceptor side (SOD and APX) are deactivated and/or degraded by excess light [8]. Thus, if the ROSs generation can be inhibited or the formed species can be scavenged before they attack the polypeptides, the integrity of PSI will be preserved. In this context, it has been reported that the improvement of the antioxidant system during *in vitro* experiments by ROSs scavengers (SOD, catalase and n-popyl gallate) can preserve the PSI structure and maintain its function against PI [15], [24]. It was reported that PAs can contribute to the improvement of the Hallywell-Asada pathway *in vivo* [58]. As mentioned above, it is shown here that PAs exert a scavenging effect against O_2_^−^ (Fig. 3). Also, it is possible that PAs can protect the antioxidant enzymes located in the PSI stromal side against photo-degradation, thus preserving their function.

Contrary to the artificial scavengers like n-propyl gallate, we may hypothesize that the biogenic PAs can contribute to a naturel strategy to enhance the antioxidant defense system. Our results provide evidence for the O_2_^−^ scavenging by Spm and Spd in photosynthetic membranes. Indeed, we observed that the PAs-O_2_^−^ scavenging action is present and exclusively ascribed to antioxidant character of these PAs at concentration of 1 mM. These results are consistent with several works which reported that PAs can directly scavenge O_2_^−^ and OH^.^, and quench chemically-generated ^1^O_2_ under *in vitro* conditions [59], [60]. Based on electron paramagnetic resonance, nuclear magnetic resonance and mass spectroscopy studies, Ha et al. [59] demonstrated that OH^.^ scavenging occurred in reactions of Spm oxidation.

Recent study reported that over-expression of PAs in thylakoid membranes stimulates the thermal dissipation of absorbed light energy in LHCII of tobacco plants [39]. The quenching of Chl fluorescence may decrease the accumulation of singlet excited state of Chl (Chls*), resulting in a drop of triplet excited states (^3^Chls*) reducing thereby the pathway for the generation of ^1^O_2_. On the other hand, Khan et al. [60] showed that Spm quenches ^1^O_2_ via a charge-transfer process to protect DNA. The rate constant for the formation ^1^O_2_-Spm is higher than that of ^1^O_2_-DNA. Similar processes may be implicated in the protection of PSI against PI.

To better assume these roles, Spm and Spd must be in a very close proximity to the sites of their action. The interaction of PAs with thylakoid membranes is likely ensured by their polycationic nature. Despite that the two PAs presented a similar pattern in protecting PSI against PI, we observed that Spm is more effective than Spd. The little difference observed between the two PAs may be attributed to the difference in their chemical properties such as the number of their positive charges (four for Spm and three for Spd). This feature allows them to interact with the negatively charged stromal side of thylakoid membranes [61]. The most positively charged PAs strongly bind to protein carboxylic groups compared to the least ones [62]. This electrostatic interaction can stabilize the protein structure, leading to the preservation of thylakoid membrane integrity and function [61], [63]. Furthermore, the electrovalent attachment of PAs to thylakoid membranes may concur to their close proximity of the sites of ROSs generation to better assume their antioxidant role.

The strong inhibition of PSII activity by PAs is shown in Fig. 4A and 6A. The inhibition of electron transfer at Cyt b_6_/f protects the photosynthetic membranes against photo-oxidative stress [64]. Similarly, when PAs fully inhibit PSII activity (7 mM), the O_2_^-^ scavenging is solely due to a decrease of electron flow towards the acceptor side of PSI. In the range of concentration between 1.5 and 7 mM a clear distinction between PSI protection against PI due to the above inhibition of superoxide formation and direct scavenging of O_2_^−^ by PAs cannot be performed. However, the greatest part of the protective action can still be attributed to PSII inhibition by the PAs. The inhibition of PSII activity observed with non-physiological doses of Spm or Spd (Fig. 4A and 6A) was considered harmful when the activity of PSII was studied separately in isolated thylakoids or PSII sub-membrane fractions [65]. But, *in vivo*, when PSII pumps electrons to the PSI complex under stress conditions, the importance of PSII deactivation must be taken into account. Especially, when the PSII repair and PAs biosynthesis are finely controlled and require PSI activity to fulfill the need for ATP. This point of view is important in the understanding of the importance of PSI resistance to photoinhibition *in vivo*.

## Conclusion

We report in this study that exogenous application of Spm or Spd on thylakoid membranes resulted in the preservation of PSI activity against PI under *in vitro* conditions. The mechanisms of PSI photo-protection minimized the PI-induced oxidative stress. This is clearly due to a direct O_2_^−^ scavenging by PAs at physiological concentration and/or due to the inhibition of electron transfer from PSII towards PSI at higher doses.

## Supporting Information

Figure S1 **Comparison of the variation of O_2_ uptake rates in control (Ctrl) and photoinhibited (PI) samples preloaded or not with 7 mM Spm.** Ctrl: control, Ctrl+7 mM Spm: control loaded with 7 mM Spm, PI: photoinhibited, PI+7 mM Spm: photoinhibited in the presence of 7 mM Spm.
(DOCX)
