# Supplementary material for: Protective Action of Spermine and Spermidine against Photoinhibition of Photosystem I in Isolated Thylakoid Membranes
Source: PLoS One. 2014 Nov 24;9(11):e112893. doi: 10.1371/journal.pone.0112893 (PMC4242612; doi:10.1371/journal.pone.0112893)
Supplement: Figure S1 — Comparison of the variation of O2 uptake rates in control (Ctrl) and photoinhibited (PI) samples preloaded or not with 7 mM Spm. Ctrl: control, Ctrl+7 mM Spm: control loaded with 7 mM Spm, PI: photoinhibited, PI+7 mM Spm: photoinhibited in the presence of 7 mM Spm. (DOCX) [file pone.0112893.s001.docx]

Supplementary information


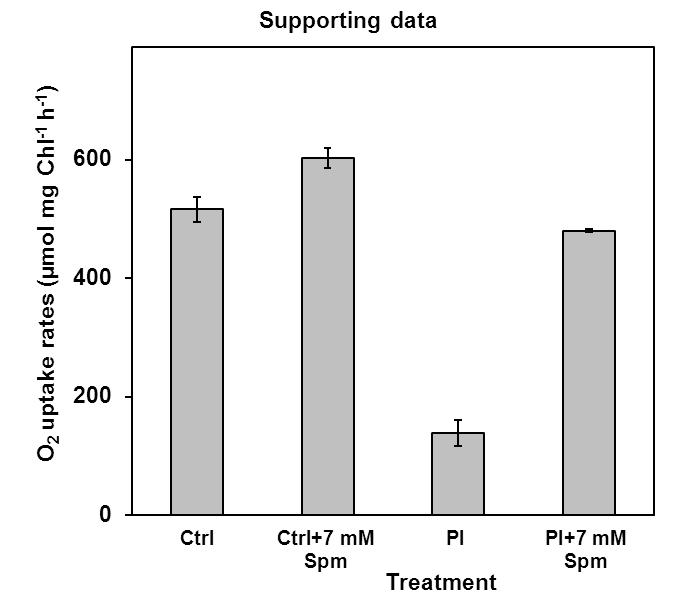


**Supporting data**: Comparison of the variation of O_2_ uptake rates in control (Ctrl) and photoinhibited (PI) samples preloaded or not with 7 mM Spm. Ctrl: control, Ctrl+7 mM Spm: control loaded with 7 mM Spm, PI: photoinhibited, PI +7 mM Spm: photoinhibited in the presence of 7 mM Spm.
